# Supplementary material for: Synergistic Antimicrobial Effect of Cold Atmospheric Plasma and Redox-Active Nanoparticles
Source: Biomedicines. 2023 Oct 13;11(10):2780. doi: 10.3390/biomedicines11102780 (PMC10604215; doi:10.3390/biomedicines11102780)
Supplement: Supplementary file 1 [file biomedicines-11-02780-s001.zip › biomedicines-2621852-supplementary.pdf]

Supplementary data

Synergistic antimicrobial effect of cold atmospheric plasma and redox-active nanoparticles

Artem M. Ermakov[1,2,3], Vera A. Afanasyeva[1,2], Alexander V. Lazukin[4], Yuri M. Shlyapnikov[2], Elizaveta S. Zhdanova[1,2], Anastasia A. Kolotova A.A.[2], Artem S. Blagodatski A.S.[2], Olga N. Ermakova O.N.[2], Nikita N. Chukavin[2,5], Vladimir K. Ivanov[6], Anton L. Popov[2,5]

[1] Hospital of the Pushchino Scientific Center of the Russian Academy of Sciences, 142290 Pushchino, Moscow Region, Russia; beoluchi@yandex.ru (A.M.E.)
[2] Institute of Theoretical and Experimental Biophysics of the Russian Academy of Sciences, 142290 Pushchino, Moscow Region, Russia; va_vera_afanaseva@mail.ru (V.A.A.), yuri.shluyapnikov@gmail.com (Y.M.S.), dla_lisa@mail.ru (E.S.Z.), anas.kolotowa2010@ya.ru (A.A.K.), bswin2000@gmail.com (A.S.B.), beoluchi@yandex.ru (O.N.E.), chukavinnik@gmail.com (N. N. C.), antonpopovleonid@gmail.com (A.L.P.)
[3] ANO Engineering Physics Institute, 142210 Serpukhov, Moscow Region, Russia; beoluchi@yandex.ru (A.M.E.)
[4] Troitsk Institute of Innovative and Thermonuclear Research (JSC «SSC RF TRINITY»), 108840, Moscow, Troitsk, Russia; lazukin@triniti.ru (A.V.L.)
[5] The State University of Education, Moscow 141014, Russia; chukavinnik@gmail.com (N. N. C.), antonpopovleonid@gmail.com (A.L.P.)
[6] Kurnakov Institute of General and Inorganic Chemistry, Russian Academy of Sciences, 119991 Moscow, Russia; van@igic.ras.ru (V.K.I.)
* Correspondence: e-mail: antonpopovleonid@gmail.com (A.L.P.)

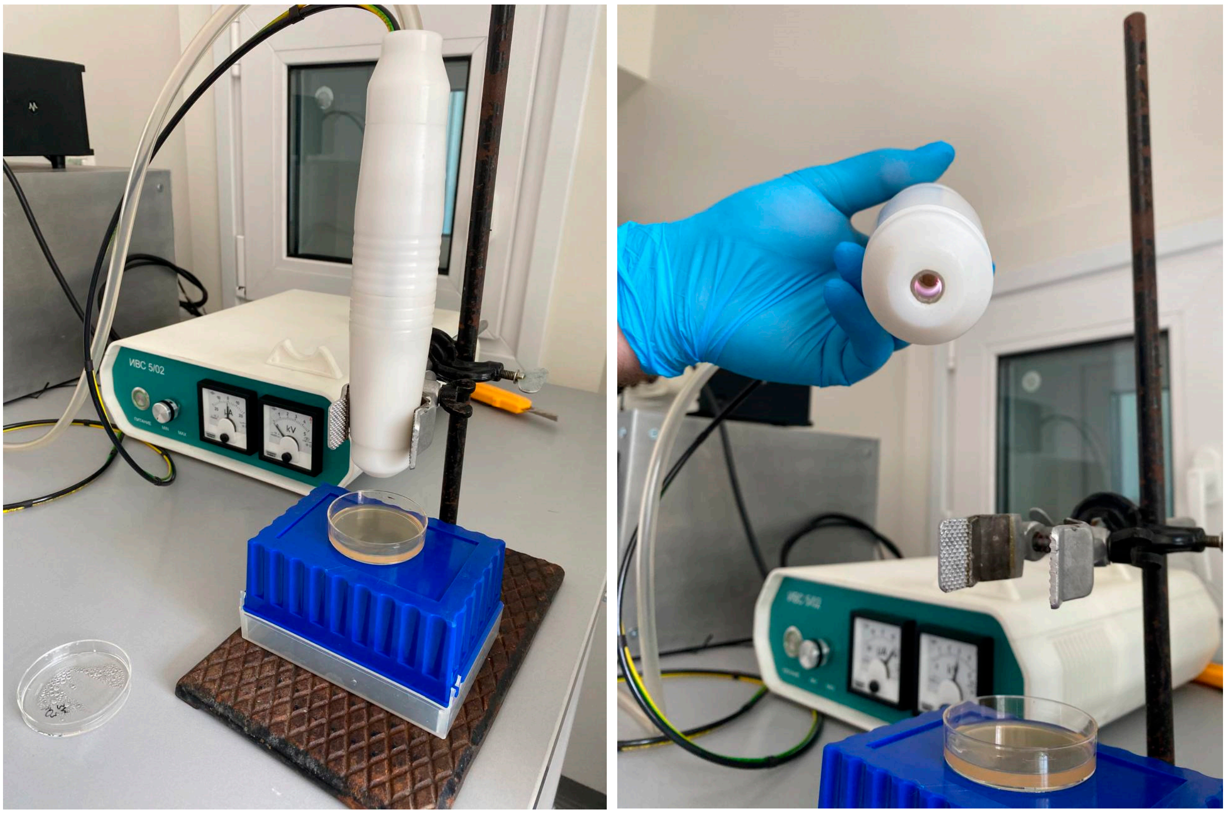

Figure S1. Irradiation scheme with the CAP irradiator (a) and demonstration of the nozzle with the outgoing plasma jet (b).

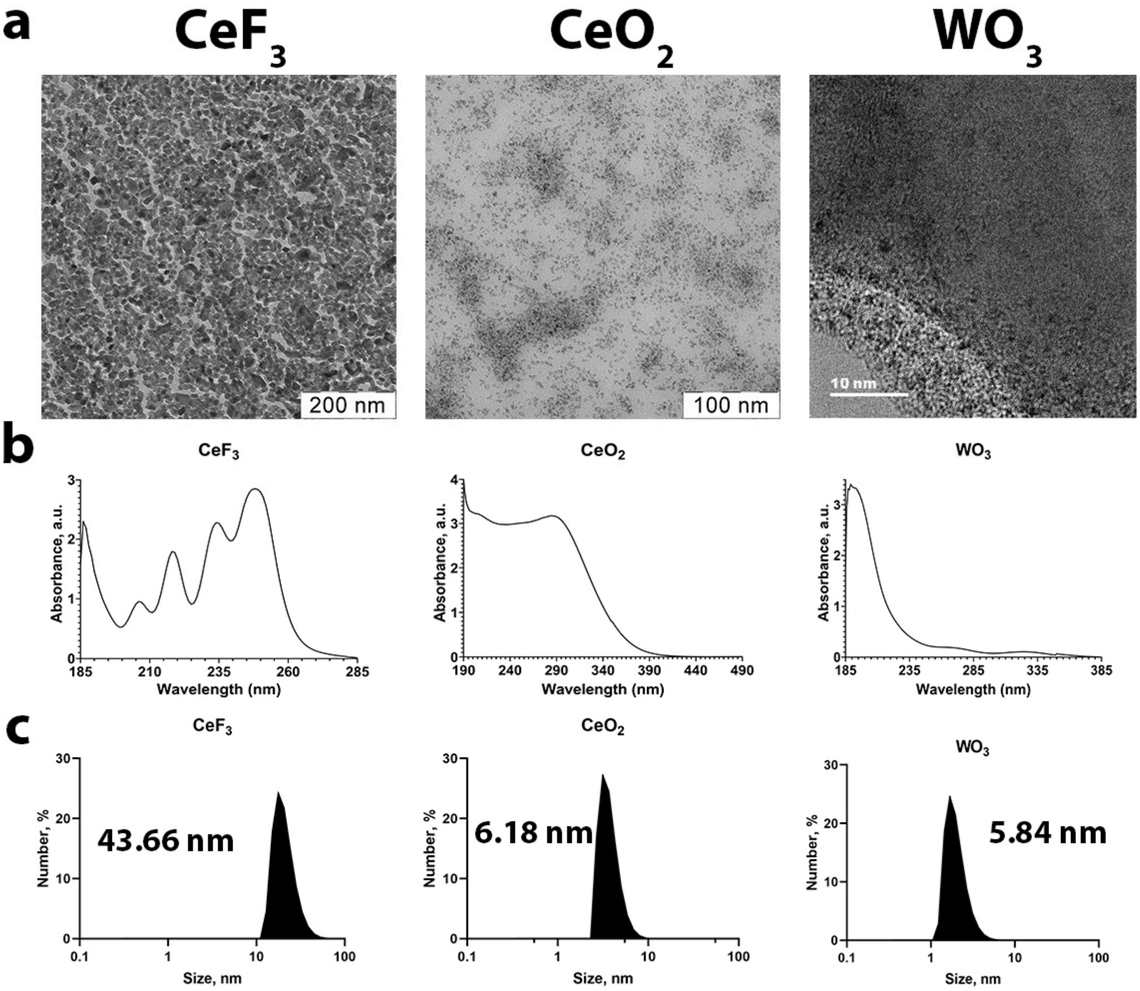

Figure S2. Characterisation of CeF_3, CeO_2 and WO_3 nanoparticles: transmission electron microscopy (a), UV/visible spectra (b) and hydrodynamic diameters after dilution in distilled water (c).

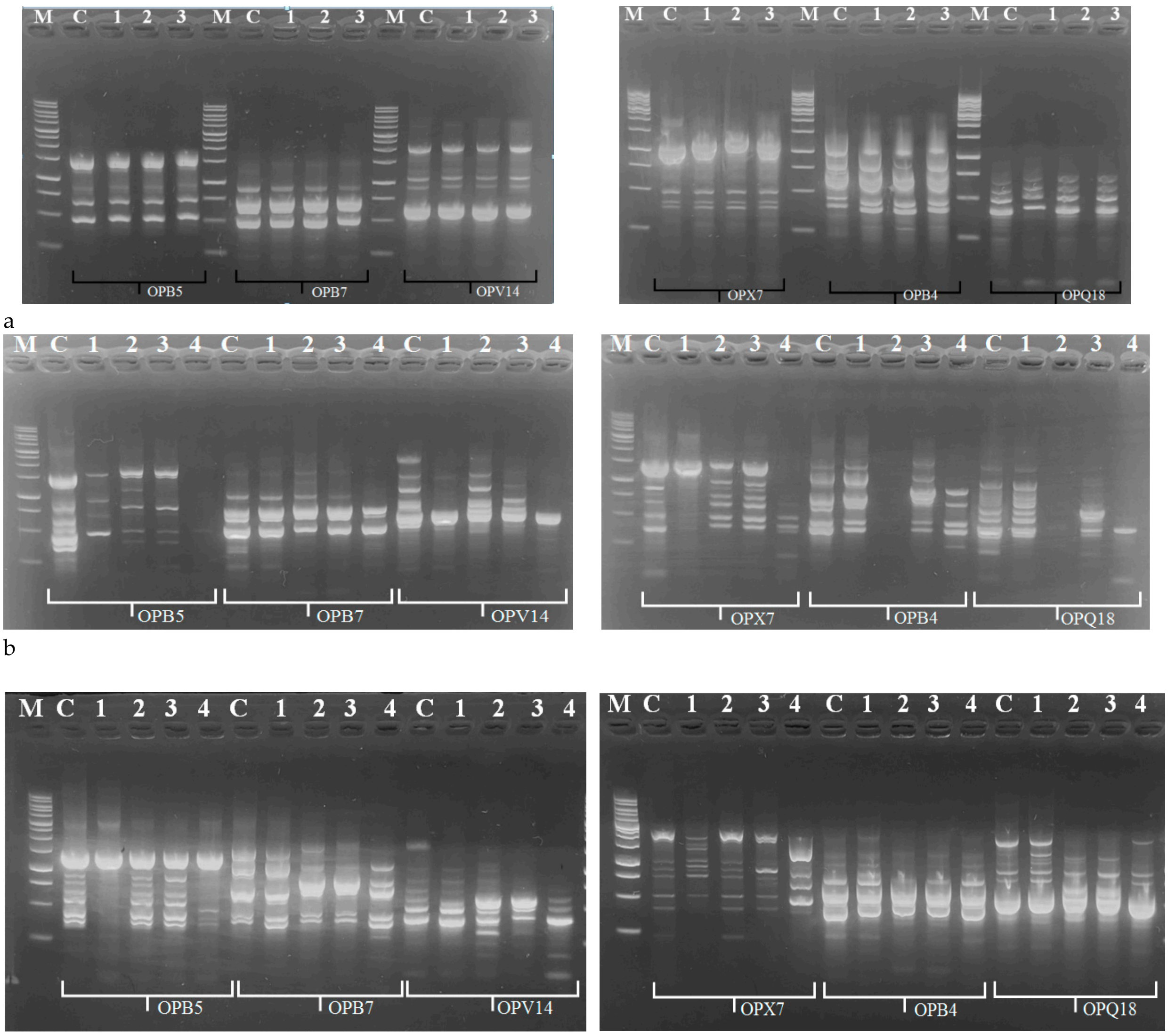

Figure S3. Results of RAPD PCR. a – samples treated with NPs, without CAP exposition. M – marker, C – control without NPs, 1- CeO_2, 2 - CeF_3, 3 - WO_3; b – samples exposed to 3 min of CAP. M – marker, C – control, 1 – no NPs, 2 - CeF_3, 3 – CeO_2, 4 - WO_3; c – samples exposed to 6 min of CAP. M – marker, C – control without NPs, 1 –no NPs, 2- CeF_3, 3 – CeO_2, 4 – WO_3.

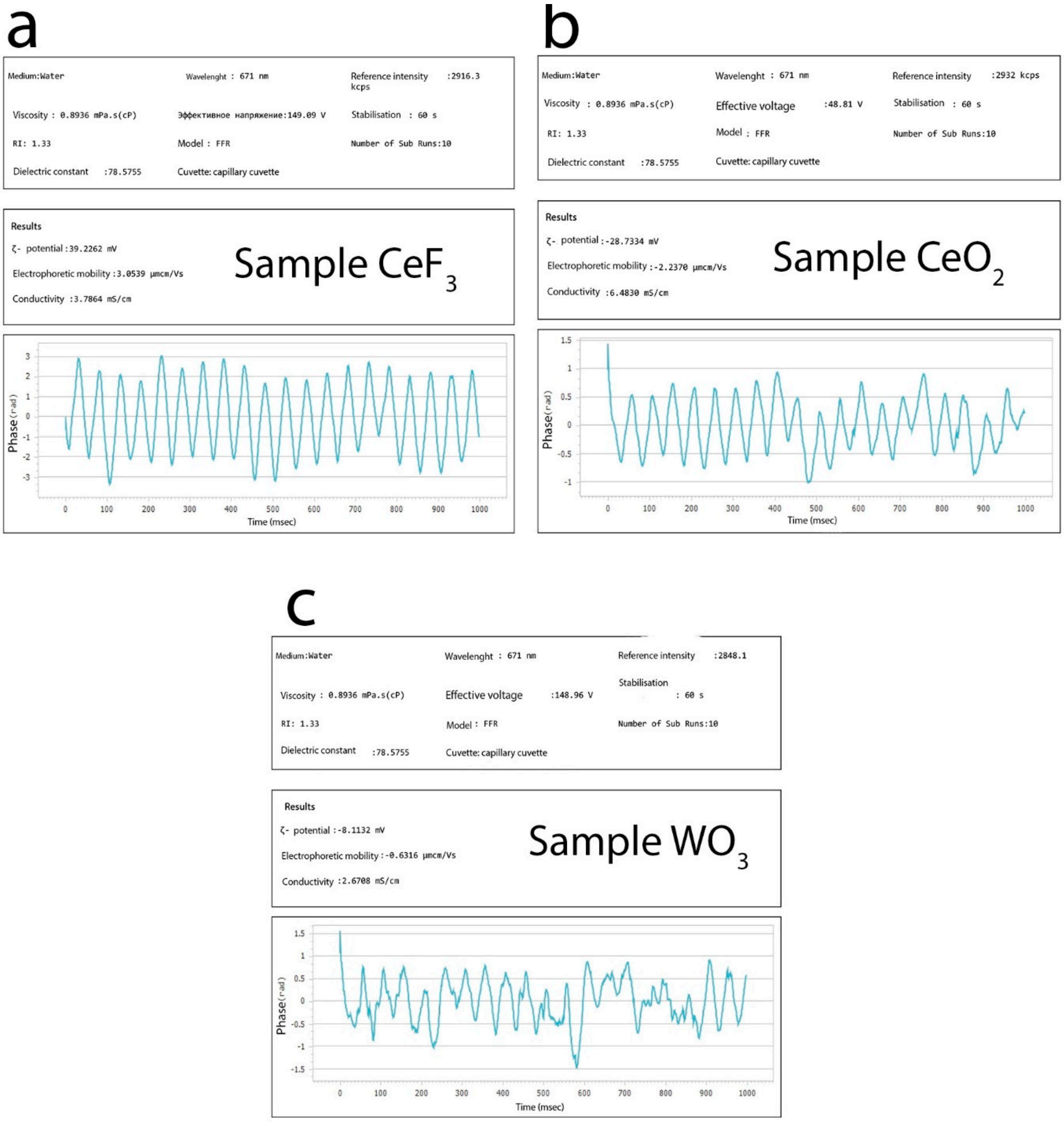

Figure S4. Zeta-potential values of the nanoparticles in distilled water: CeF_3 (a), CeO_2 (b), WO_3 (c).
